# 5-Aza-2′-deoxycytidine advances the epithelial–mesenchymal transition of breast cancer cells by demethylating *Sipa1* promoter-proximal elements

**DOI:** 10.1242/jcs.236125

**Published:** 2020-05-11

**Authors:** Ang Lu, Wei Wang, Shu-Fang Wang-Renault, Brian Z. Ring, Yoshimasa Tanaka, Jun Weng, Li Su

**Affiliations:** 1 Key Laboratory of Molecular Biophysics of Ministry of Education, College of Life Science and Technology, Huazhong University of Science and Technology, Wuhan, 430074, China; 2 INSERM UMR-S1147, CNRS SNC5014; Paris Descartes University, Equipe Labellisée Ligue Nationale Contre le Cancer, Paris 75006, France; 3 Institute of Genomic and Personalized Medicine, College of Life Science and Technology, Huazhong University of Science and Technology, Wuhan, Hubei 430074, China; 4 Center for Medical Innovation, Nagasaki University, 1-7-1, Sakamoto, Nagasaki, 852-8588, Japan; 5 Research Institute of Huazhong University of Science and Technology in Shenzhen, Shenzhen, 518063, China

**Keywords:** 5-Aza-CdR, Hypomethylation, *Sipa1*, Breast cancer, Epithelial–mesenchymal transition

## Abstract

Human breast cancer cells exhibit considerable diversity in the methylation status of genomic DNA CpGs that regulate metastatic transcriptome networks. In this study, we identified human *Sipa1* promoter-proximal elements that contained a CpG island and demonstrated that the methylation status of the CpG island was inversely correlated with SIPA1 protein expression in cancer cells. 5-Aza-2′-deoxycytidine (5-Aza-CdR), a DNA methyltransferase inhibitor, promoted the expression of *Sipa1* in the MCF7 breast cancer cells with a low level of SIPA1 expression. On the contrary, in MDA-MB-231 breast cancer cells with high SIPA1 expression levels, hypermethylation of the CpG island negatively regulated the transcription of *Sipa1*. In addition, the epithelial–mesenchymal transition (EMT) was reversed after knocking down *Sipa1* in MDA-MB-231 cells. However, the EMT was promoted in MCF7 cells with over-expression of SIPA1 or treated with 5-Aza-CdR. Taken together, hypomethylation of the CpG island in *Sipa1* promoter-proximal elements could enhance SIPA1 expression in breast cancer cells, which could facilitate EMT of cancer cells, possibly increasing a risk of cancer cell metastasis in individuals treated with 5-Aza-CdR.

## Abstract (precis)

**Summary:** Hypomethylation by 5-Aza-CdR upregulates the SIPA1 expression and promotes epithelial–mesenchymal transition in breast cancer cells, possibly increasing the risk of cancer cell metastasis.

## INTRODUCTION

In mammalian cells, the methylation of cytosine residues in genomic DNA is an epigenetic modification that can play an important role in the control of gene expression (Ehrlich et al., 1982). The modification of cytosines takes place after DNA replication and is catalyzed by DNA methyltransferase to generate 5-methylcytosines. In malignant cells, the methylation status of the CpG island in the promoter region of many cancer-related genes, such as tumor suppressor genes and genes that suppress metastasis and angiogenesis is frequently altered, resulting in the silencing of their expression (Baylin et al., 2001; Esteller and Herman, 2002; Jones and Baylin, 2002; Issa, 2004; Ding et al., 2018). Reversal of gene suppression by the inhibition of DNA methyltransferases has been successful in the treatment of benign and malignant cells. 5-Aza-2′-deoxycytidine (5-Aza-CdR), a specific inhibitor of DNA methylation, has been demonstrated to reverse the inhibition of many tumor suppressor genes in human tumor cell lines. Many *in vitro* experiments and clinical trials have shown promising results of 5-Aza-CdR therapy in the treatment of many malignancies, including myeloid leukemias (Wijermans et al., 2000; Chuang et al., 2005; Roulois et al., 2015).

In breast cancers, DNA methylation does not occur randomly and definitive patterns are associated with clinically and biologically relevant subtypes (Stefansson et al., 2015; The Cancer Genome Atlas Network, 2012). DNA methylation occurs almost exclusively in a symmetric CG context and many of the genes containing the CpG island make up metastatic transcriptomes. The methylation status of the CpG island thus accounts for a transcriptomal diversity found in breast cancers with varying prognosis, indicating a fundamental epigenomic contribution to metastasis (Fang et al., 2011). SIPA1, signal-induced proliferation-associated protein 1, promotes breast cancer cell invasion, migration and metastasis (Park et al., 2005; Zhang et al., 2015). SIPA1 was originally found to be highly expressed in human lymphoid tissues, including the spleen, thymus and peripheral blood leukocytes (Kurachi et al., 1997). In addition to lymphoid tissues, a high level of SIPA1 expression was also observed in the hippocampus, myocardial cells and skeletal muscle, whereas only marginal or no expression of SIPA1 was found in the skin, breast, prostate and gastrointestinal tract (Uhlen et al., 2015). Previous studies have shown that SIPA1 is closely associated with the adhesion, invasion and metastasis of tumor cells (Tsukamoto et al., 1999; Park et al., 2005). SIPA1 reduced the adhesion of HeLa cells by interacting with AF-6, a cytoskeleton-anchoring protein (Su et al., 2003). In some cancerous tissues such as the breast, prostate and colon, a markedly high level of SIPA1 is expressed, compared with that in the surrounding normal tissues, that may be responsible for cancer metastasis (Minato and Hattori, 2009; Zhang et al., 2015; Shimizu et al., 2011; Ji et al., 2012). Through interacting with Rap1b/Brd4 to form a metastatic transcriptomal network, SIPA1 regulates the expression of extracellular matrix genes (Alsarraj et al., 2013; Crawford et al., 2008; Farina et al., 2004). Our previous work demonstrated that nuclear-localized SIPA1 interacted with the promoter of the integrin β1 gene and induced its transcription, possibly promoting the breast cancer invasion (Zhang et al., 2015).

SIPA1 is, however, not always highly expressed in all breast cancer cells and other malignant cells, e.g. only a low level of SIPA1 is expressed in the breast cancer cell line MCF7 (Zhang et al., 2015). In addition, we demonstrate in the present study that various cancer cell lines express SIPA1 to different degrees. The mechanism underlying the marked diversity in SIPA1 expression has not yet been reported.

It is well known that DNA hypomethylation is one of the major epigenetic abnormalities associated with a wide variety of cancer phenotypes and can occur over widespread chromosomal regions or at discrete loci. A recent study showed that soluble factors secreted from cancer-associated fibroblasts could upregulate the transcription of certain genes with hypermethylated CpG island in human breast tumors (Mathot et al., 2017). Tobacco smoking also had a significant effect on DNA methylation. In fact, smokers exhibited 1.5% lower methylation of the *Sipa1* gene at the 5′-UTR region than individuals who had never smoked (Steenaard et al., 2015). The human SIPA1 protein is encoded by the *Sipa1*, which is localized to 11q13 with 16 exons and 15 introns, but the fine structure of the promoter region remains unclear. In the present study, we identified the promoter-proximal elements of the *Sipa1* that contained a CpG island. To explore the mechanism underlying the dysregulation of SIPA1 expression, the effect of 5-Aza-CdR on the demethylation of the CpG island and the subsequent cellular alterations were investigated.

## RESULTS

### SIPA1 expression varies in different cancer cells

**Fig. 1.**
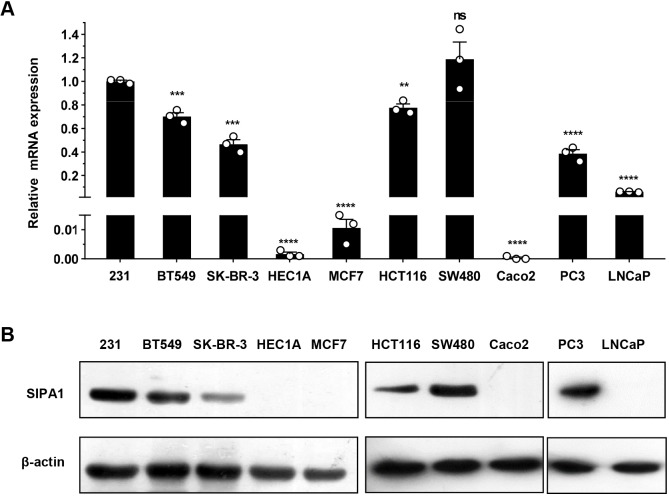
**SIPA1 expression exhibits heterogeneity among human cancer cell lines.** (A) mRNA levels of the *Sipa1* gene in breast, colon, endometrial and prostate cancer cells, as determined by qRT-PCR. Data are means±s.d. and are representative of three independent experiments. **P<0.01, ***P<0.001, ****P<0.0001; ns, no significance; two-tailed unpaired Student's *t*-test. (B) The expression level of SIPA1 protein in 10 different cancer cell lines by western blotting. ‘231’ represents the MDA-MB-231 cell line.

To analyze the expression of SIPA1 in different cancer cell lines, we determined the mRNA levels of the *Sipa1* by quantitative real time-PCR (qRT-PCR) in four breast cancer cell lines (MDA-MB-231, BT549, SK-BR-3 and MCF7), three colon cancer cell lines (HCT116, SW480 and Caco2), two prostate cancer cell lines (PC3 and LNCaP) and an endometrial adenocarcinoma cell line (HEC1A). As shown in Fig. 1A, 10 cancer cell lines expressed the *Sipa1* mRNA to different degrees. We then compared SIPA1 protein expression among the above cancer cell lines. SIPA1 protein levels were significantly higher in MDA-MB-231, BT549, SK-BR-3, HCT116, SW480 and PC3 cell lines, whereas the protein was hardly detected in MCF7, HEC1A, Caco2 and LNCaP cell lines (Fig. 1B), which was consistent with the results obtained by qRT-PCR analyses. The SIPA1 protein was undetectable in MCF7 cells with low migration capacity. By contrast, relatively high levels of SIPA1 protein were observed in the other three breast cancer cell lines (MDA-MB-231, BT549 and SK-BR-3) with high migration capacity (Zhang et al., 2015; Chen et al., 2018; Ma et al., 2017).

### Methylation status of the CpG island in the *Sipa1* promoter-proximal elements is inversely correlated with SIPA1 expression in cancer cells

**Fig. 2.**
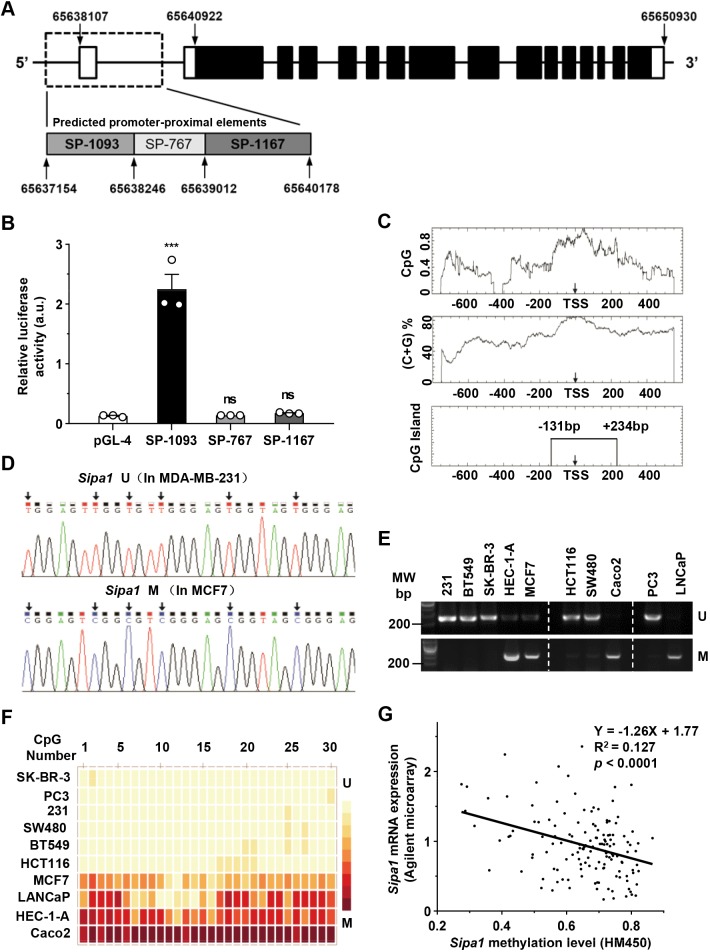
**Methylation status of the CpG island in *Sipa1* promoter-proximal elements is inversely correlated with *Sipa1* mRNA expression.** (A) A schematic of the *Sipa1* gene with the three possible promoter-proximal elements. The numbers represent the nucleotide position in the genome sequence of chromosome 11. The boxes on the main chain represent exons. The lines connecting the boxes represent introns. The black filled boxes represent coding sequences; the empty unfilled boxes represent UTRs. (B) Identification of the sequence containing *Sipa1* promoter-proximal elements assessed using a luciferase assay. The respective plasmids and pGL-4 as a control were co-transfected into HEK293 cells with pRL-TK. The numbers represent the average luminescence intensity per sample in an assay performed in triplicate. ***P<0.001; ns, no significance; two-tailed unpaired Student's *t*-test. Error bars indicate the s.e.m. (C) Prediction of the CpG island in *Sipa1* promoter proximal elements by CpGPlot. The criteria for prediction were an observed/expected ratio of more than 0.6, percent (C+G) or more than 50% and a length greater than 200 bp. (D) Sequencing validation of the methylation-specific PCR (MSP) sensitivity. The PCR products from MDA-MB-231 and MCF7 cells were purified and sequenced. The positions of the converted cytosines in the CpG dinucleotides are indicated by arrows. (E) MSP of the *Sipa1* CpG island in 10 different cancer cell lines. ‘U’ represents unmethylated nucleotides; ‘M’ represents methylated nucleotides. (F) Heatmap depicting the proportion of methylated CpG dinucleotides within the *Sipa1* CpG island in 10 cancer cell lines. Each box indicates one of 30 individual CpG dinucleotides. The proportion is represented by the darkness of the red color; the methylation ranged from 0% to 100%. ‘231’ represents the MDA-MB-231 cell line. (G) The correlation analysis between the mRNA level and the methylation level of the *Sipa1* gene in human breast tumor samples from TCGA breast cancer tissues data set. A linear regression analysis was performed to evaluate the correlation between the two factors.

First, we set out to locate promoter-proximal elements that regulated the expression of *Sipa1* in human genomic DNA. According to the prediction made by SwitchGear Genomics, a possible DNA sequence that regulated *Sipa1* gene was the 1093-nucleotide (nt) segment from 65637154 to 65638246 in the genome sequence of chromosome11 (from GRCh38.p7/hg38), as shown in Fig. 2A. This segment contained a transcription start site (TSS). The alternative promoter-like region predicted by the same program was the 1167-nt segment from 65639012 to 65640178. There was a gap between the two predicted promoter-proximal elements, which contained 767 nucleotides, and we also consider this segment to contain the *Sipa1* promoter-proximal elements. These three nucleotide segments were termed as SP-1093, SP-1167 and SP-767, according to the segment lengths, respectively. By using several promoter prediction programs, we confirmed that SP-1093 had the highest probability of being the *Sipa1* promoter-proximal elements among the three segments (Tables S1-S3). To evaluate the promoter prediction, the three DNA segments were cloned into the pGL-4 vector with the luciferase gene. Using a dual-luciferase reporter assay system, only SP-1093 induced the expression of downstream luciferase (Fig. 2B), indicating that SP-1093 was most likely to contain the promoter–proximal elements of *Sipa1*.

It was, however, not clear whether SP-1093 covered the whole promoter-proximal elements or only part of them. Based on an *in silico* prediction, a CpG-rich sequence existed in the predicted *Sipa1* promoter-proximal elements, which was mostly located in the SP-1093 region but also extended into SP-767. To confirm this possible CpG island, a 1400-nt sequence near the TSS (−800 nt to +600 nt) was analyzed using an EMBOSS CpGPlot program (Fig. 2C). The result suggested the existence of a 325-bp potential CpG island between −131 bp and +234 bp, which was consistent with the prediction made by a MethPrimer software (data not shown). To examine the possible function of the entire CpG island region as part of the *Sipa1* promoter-proximal elements, we designed an extended construct, SP-1203, flanked with a 3′ terminus that was overlapped with the 5′ stretch of the SP-767 sequence to include the whole predicted CpG island, and several deletion constructs, as shown in supplementary information (Fig. S1A). With the use of a dual-luciferase reporter assay system, we found that the promoter activity of SP-1203 was slightly lower than that of SP-1093, with no statistically significant difference. All deletion segments without the CpG-rich stretch failed to exhibit promoter function (Fig. S1B), indicating that SP-1093 contained the functional promoter-proximal elements of *Sipa1* and that this CpG region was essential for the transcription of *Sipa1*.

It is well known that the methylation status of the CpG island within gene promoter-proximal elements affects gene transcription, and dysregulated methylation is often observed in cancer-related genes of malignant tumors (Deaton and Bird, 2011). By sequencing the methylation-specific PCR (MSP) products of the CpG island in *Sipa1* promoter region, we found that CpG dinucleotides were unmethylated in MDA-MB-231 cells and methylated in MCF7 cells, which is consistent with the expression status of SIPA1 in the two cell lines (Fig. 2D). Furthermore, we compared the methylation status of the CpG island within the *Sipa1* promoter-proximal elements among ten different tumor cell lines by MSP. Of these 10 cell lines, MDA-MB-231, BT549, SK-BR-3, HCT-116, SW480 and PC3 exhibited unmethylated PCR bands, but not methylated bands. On the other hand, the other four cell lines, HEC1A, MCF7, Caco2 and LNCaP, showed methylated PCR bands (Fig. 2E). The former cell lines are highly metastatic and the latter cell lines are not. Using bisulfite sequencing PCR (BSP), genomic fragments covering the entire CpG island of the 10 cancer cell lines were amplified and sequenced (see Fig. S2 for the original methylation status of CpG sites). The proportions of methylated CpG dinucleotides in each cell line were then plotted with a color gradient according to methylation levels on a heatmap. As shown in Fig. 2F, the *Sipa1* CpG regions of the tumor cell lines were methylated to different degrees. For example, in MDA-MB-231, HCT116 and PC3, the CpG segments were not extensively methylated and *Sipa1* transcription levels were relatively high. On the other hand, the methylation levels were significantly high in Caco2 and LNCaP, which exhibited low *Sipa1* transcription. We also examined the relationship between the *Sipa1* methylation status and the mRNA expression level in the TCGA breast cancer dataset. As shown in Fig. 2G, the *Sipa1* mRNA expression is negatively correlated with the methylation level of the CpG island. Furthermore, we made comparisons between the methylation status of the *Sipa1* promoter region in human tissues and cancer cell lines from the GEO whole-genome bisulfite sequencing dataset, and *Sipa1* transcription levels in human tissues and cancer cell lines from Human Protein Atlas. We found that CpG methylation status was negatively correlated with SIPA1 expression in human tissues as well as cancer cell lines (Fig. S3). Taken together, the *Sipa1* mRNA expression might be inversely correlated with the methylation status of the CpG island in the *Sipa1* promoter-proximal elements of cancer cells, especially in breast cancer cells.

### Methylation of the CpG island reduces the transcription of *Sipa1*

**Fig. 3.**
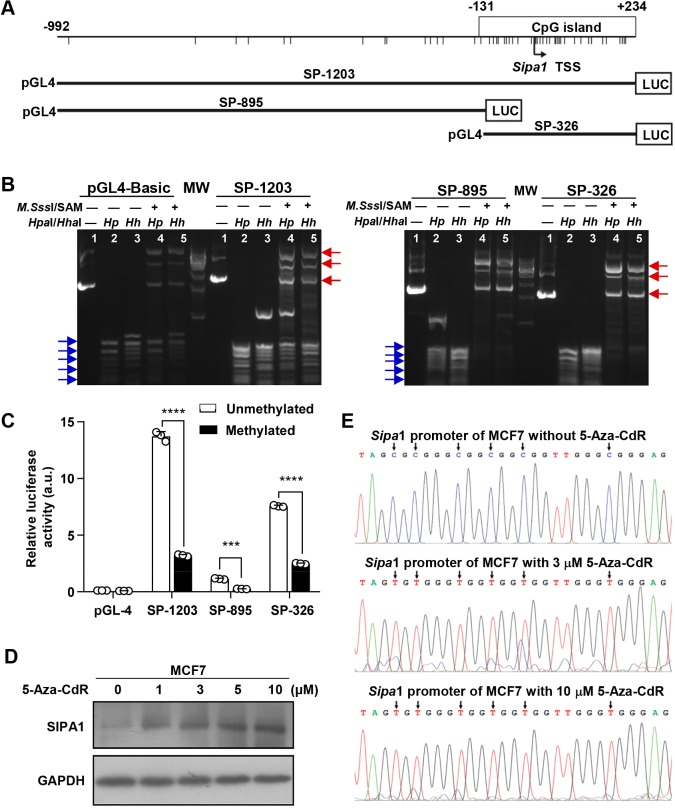
**Methylation status of the CpG island stipulates *Sipa1* transcription in breast cancer cells.** (A) *Sipa1* promoter plasmid construction. In the map of the *Sipa1* promoter proximal elements, the CpG dinucleotide is indicated by short vertical lines and the CpG island is represented by a rectangle. Three pGL4 plasmids containing different fragments of the possible *Sipa1* promoter-proximal elements: SP-1203 contains the entire promoter sequence, SP-895 lacks the CpG island and SP-326 contains the CpG island only. (B) Methylation status of each plasmid examined by digestion with methylation-sensitive restriction enzymes. Lane 1 is a negative control; lanes 2 and 3 represent plasmids treated with *M*.*Sss*I without SAM as methylation substrates and digested with methylation-sensitive restriction enzyme HpaI (*Hp*) or HhaI (*Hp*); lanes 4 and 5 represent plasmids treated with *M*.*Sss*I and SAM, and digested with HpaI (*Hp*) or HhaI (*Hp*). Blue arrows indicate the digested DNA bands that were sensitive to HpaI or HhaI digestion; red arrows indicate the DNA bands that were more resistant to HpaI or HhaI digestion. (C) Transcriptional activity of the possible *Sipa1* promoter-proximal elements with varying degrees of methylation status. Each plasmid was transfected into MDA-MB-231 cells, and the luciferase activity was measured 36 h later. The pGL4-Basic plasmid was used as a negative control. The columns represent the mean of triplicate experiments (***P<0.001, ***P<0.0001, two-tailed unpaired Student's *t*-test. Error bars indicate the s.e.m.). (D) Induction of SIPA1 expression in MCF7 cells treated with different concentrations of 5-Aza-CdR. Cells pre-treated with DMSO were used as a negative control. (E) Sequencing validation of the methylation status of *Sipa1* promoter proximal elements in MCF7 cells treated with 5-Aza-CdR. The PCR products derived from MCF7 cells with or without 5-Aza-CdR treatment were purified and sequenced. The positions of the converted cytosines in the CpG dinucleotides are indicated by arrows.

To determine whether the upregulation of SIPA1 was due to the hypomethylation of the CpG island within *Sipa1* promoter-proximal elements, we examined the promoter activity of the three DNA stretches, i.e. SP-1203, SP-895 (the promoter-proximal elements excluding the CpG island) and SP-326 [the DNA fragments with the CpG island sequence only (−131 bp to +211 bp)], as depicted in Fig. 3A. SP-1203, SP-895 and SP-326 were then methylated *in vitro* by using CpG methyltransferase *M.Sss*I in the presence of S-adenosylmethionine (SAM), and the methylation status was examined by digestion with the methylation-sensitive restriction enzyme HpaI or HhaI. As shown in Fig. 3B, without the methylation treatment, the plasmids were digested into short fragments by HpaI or HhaI. By contrast, the plasmids were digested into much larger fragments after the methylation treatment. These results indicated that the methylation levels of the DNA fragments (SP1203, SP-895 or SP-326) were low before the enzymatic methylation and the plasmids were sensitive to HpaI or HhaI digestion, whereas the enzymatic methylation rendered the plasmids more resistant to HpaI and HhaI digestion. We then compared the transcriptional activities of MDA-MB-231 cells transiently expressing the methylated plasmids with those of cells expressing unmethylated ones. The unmethylated SP-1203, possibly containing the full sequence of *Sipa*1 promoter-proximal elements, exhibited the highest promoter activity among three unmethylated plasmids. SP-895 without the CpG sequence displayed marginal promoter activity, which was only 8.2% of the SP-1203 activity, whereas SP-326, consisting of the CpG island only, showed a significant level of promoter activity, which attained up to 57% of the SP-1203 activity (Fig. 3C). The results demonstrated that the CpG island played an essential role in the transcription of *Sipa1*. When MDA-MB-231 cells were transformed with the methylated plasmids, however, the promoter activities were significantly reduced, especially for the plasmids with SP-1203 and SP-326, which contained the CpG island sequence (Fig. 3C). In summary, the hypermethylation of the CpG island in the possible promoter-proximal elements resulted in the downregulation of *Sipa1* transcription.

To further examine the relationship between the methylation status and the SIPA1 expression, we treated MCF7 cells with 5-Aza-CdR. It was demonstrated by western blotting analysis that the SIPA1 protein expression increased with increasing the concentration of 5-Aza-CdR (Fig. 3D). Sequencing analysis also confirmed that 5-Aza-CdR demethylated the possible *Sipa1* promoter-proximal elements at a concentration of 10 μM (Fig. 3E). Taken together, it is most likely that the methylation status of *Sipa1* is inexorably linked to the regulation of SIPA1 expression in cancer cells.

### Downregulation of *Sipa1* suppresses EMT of MDA-MB-231 cells

**Fig. 4.**
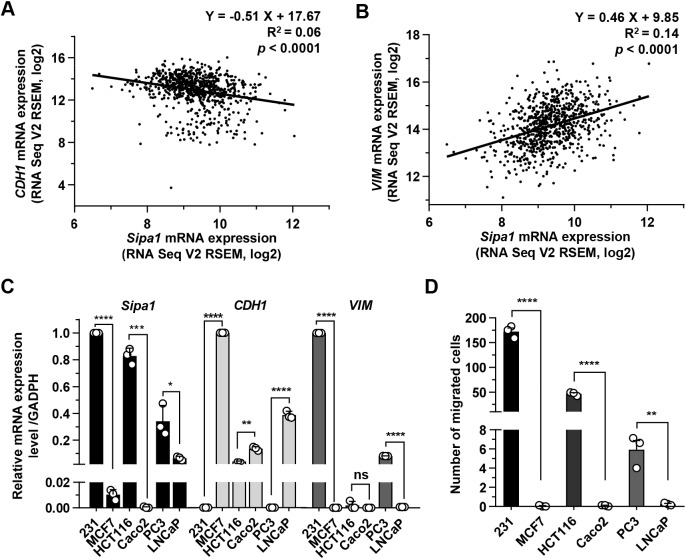
**SIPA1 expression is positively correlated with EMT of cancer cells.** (A,B) Correlation analysis of mRNA expression between *Sipa1* and *CDH1* (E-cadherin) (A), and between *Sipa1* and *VIM* (vimentin) (B) in human breast tumor samples from a TCGA breast cancer tissues dataset. The gene expression data for scatterplot were log2 transformed from TCGA mRNA expression data. A linear regression analysis was performed to evaluate the correlation between two factors. (C) mRNA levels of *Sipa1*, *CDH1* and *VIM* in six different cancer cell lines determined through qRT-PCR and normalized to the endogenous control GAPDH. Each column represents the means±s.d. of triplicate experiments. (D) The migration ability of different tumor cell lines. Cell migration was examined using a transwell migration assay. ‘231’ represents the MDA-MB-231 cell line. Columns represent the mean of triplicate experiments. *P<0.05, **P<0.01, ***P<0.001, ****P<0.0001; ns, no significance; two-tailed unpaired Student's *t*-test. Error bars indicate s.d.

Epithelial-mesenchymal transition (EMT) is closely correlated with metastatic features of tumor cells (Lambert et al., 2017). Our previous studies revealed that a high level of SIPA1 expression could promote the metastasis of breast cancer cells (Zhang et al., 2015). The finding prompted us to compare the expression patterns of SIPA1 with two molecules, vimentin and E-cadherin, which are closely associated with epithelial and mesenchymal features, in TCGA breast cancer patient samples. A linear regression analysis revealed a negative correlation between SIPA1 and E-cadherin expression in breast cancer tissues (*P<0.001) and a positive correlation between SIPA1 and vimentin expression (*P<0.001) (Fig. 4A,B). We next compared the expression patterns of *VIM* (vimentin) and *CDH1* (E-cadherin) with those of *Sipa1* using qRT-PCR in the cancer cell lines studied herein. In MDA-MB-231 and PC3 cells, a high level of SIPA1 and vimentin expression was observed, whereas E-cadherin expression was marginal (Fig. 4C). HCT116 cells expressed a high level of SIPA1, but a low level of E-cadherin and vimentin as reported previously (Kim et al., 2013; Lee et al., 2018). In contrast, the MCF7, Caco2 and LNCaP cells were characterized by low or even undetectable expression of SIPA1 but high E-cadherin and low vimentin expression (Fig. 4C). We next quantified the migration capacity of tumor cells by using a transwell migration assay system. As shown in Fig. 4D, MDA-MB-231, HCT116 and PC3 expressing a high level of SIPA1 exhibited higher migration capacity than MCF7, Caco2 and LNCaP with a marginal level of SIPA1 expression. Based on these findings, it is likely that a high level of SIPA1 expression is correlated with mesenchymal features of cancer cells, whereas a low level of SIPA1 expression is associated with epithelial features of cancer cells.

**Fig. 5.**
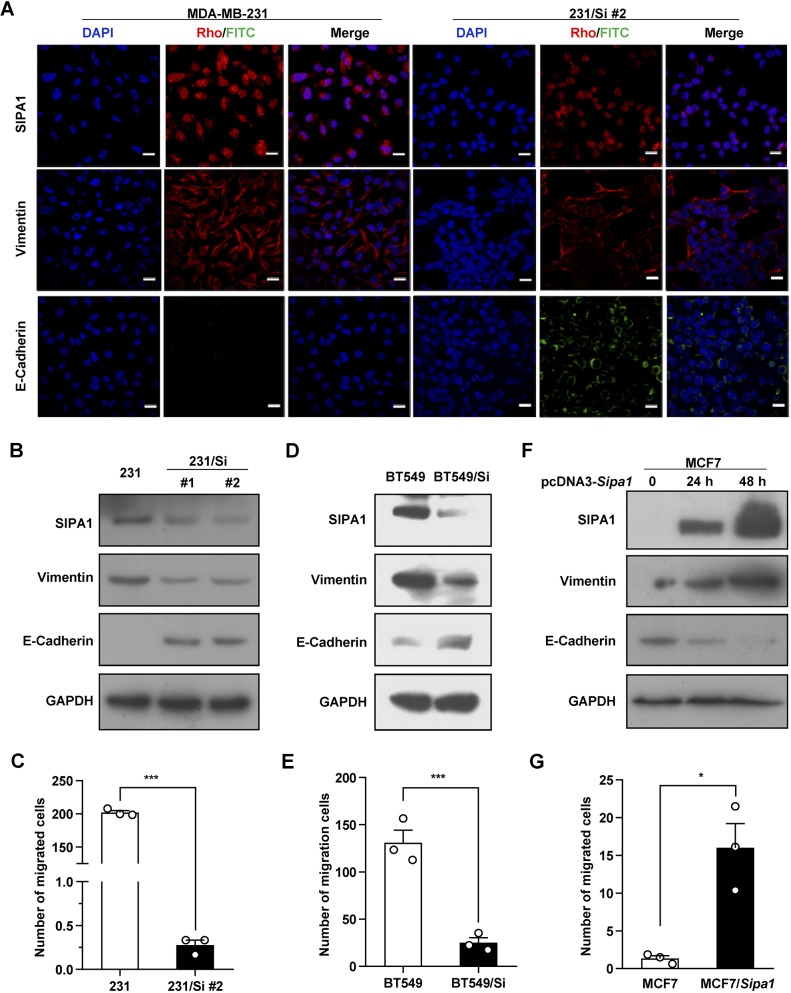
**SIPA1 promotes EMT of breast cancer cells.** (A) The effect of *Sipa1* knockdown on the expression of SIPA1 (Rho in red color), E-cadherin (FITC in green color) or vimentin (Rho in red color) in MDA-MB-231 cells (DAPI in blue color) by confocal imaging. Scale bars: 20 μm. ‘231/Si’ represents the MDA-MB-231/sh-*Sipa1* cell line. (B,D) Effect of SIPA1 knockdown on the expression of E-cadherin and vimentin in MDA-MB-231 cells (B) and in BT549 cells (D) revealed by western blotting. (C,E) Effect of SIPA1 knockdown on the cell migration of MDA-MB-231 (C) and in BT549 cells (E) measured through a transwell migration assay. ‘BT549/Si’ represents the BT549/sh-*Sipa1* cell line. ***P<0.001, two-tailed unpaired Student's *t*-test. Error bars indicate s.d. (F) The expression of SIPA1, E-cadherin and vimentin in MCF7 cells with overexpression of SIPA1 by western blotting. (G) Effect of SIPA1 overexpression on the migration of MCF7 cells. The migrated cells were counted at 24 h using a transwell assay. Each column represents the mean of triplicate experiments *P<0.05, two-tailed unpaired Student's *t*-test. Error bars indicate s.d.

We then examined the relationship between SIPA1 protein expression and EMT in breast cancer cell lines. First, the expression status of SIPA1, E-cadherin and vimentin was examined by immunofluorescence imaging under a confocal microscope. As shown in Fig. 5A, E-cadherin expression was induced and vimentin expression was suppressed in *Sipa1*-knockdown MDA-MB-231 cells, suggesting that SIPA1 seemed to be essential to maintain the mesenchymal features. Western blot analysis also confirmed increased E-cadherin expression and decreased vimentin expression in two independent *Sipa1*-knockdown MDA-MB-231 cell lines 1 and 2, compared with the parent cells (Fig. 5B). The migration of the *Sipa1*-knockdown MDA-MB-231 cell line 2 was markedly suppressed, compared with that of parent MDA-MB-231 (Fig. 5C). Furthermore, E-cadherin expression was induced and vimentin expression was suppressed in *Sipa1*-knockdown BT549 cells (Fig. 5D), and the migration of *Sipa1*-knockdown BT549 cells was suppressed compared with that of parent BT549 cells (Fig. 5E). When SIPA1 was expressed in MCF7 cells, vimentin expression was induced *de novo*, whereas E-cadherin expression was reduced in a time-dependent manner (Fig. 5F). We also observed that the migration of MCF7 was enhanced when SIPA1 was highly expressed (Fig. 5G). With overexpression of SIPA1 in BT549 cells, vimentin expression was enhanced, while E-cadherin expression was not detected by western blotting (Fig. S4). The results suggest that EMT is suppressed in breast cancer cells with a low level of SIPA1 expression.

### 5-Aza-CdR advances EMT in MCF7 cells via hypomethylation of *Sipa1*

**Fig. 6.**
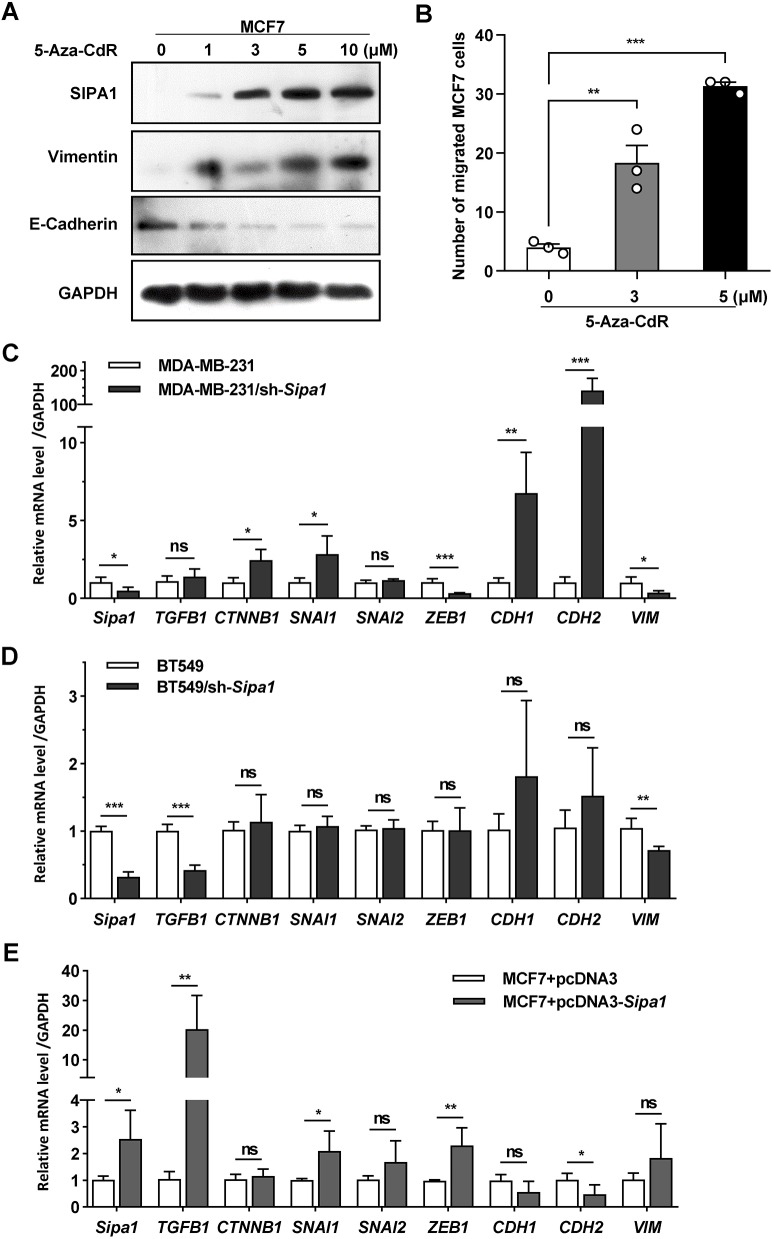
**5-Aza-CdR advances EMT in MCF7 cells via hypomethylation of *Sipa1*.** (A) Effect of 5-Aza-CdR on the expression of SIPA1, E-cadherin and vimentin in MCF7 cells. The cells were treated with increasing concentrations of 5-Aza-CdR and the protein expression was determined by western blotting. (B) Effect of 5-Aza-CdR on the migration of MCF7 cells. The cells were treated with different concentrations of 5-Aza-CdR and the migrated cells were counted by the transwell assay. Each column represents the mean of triplicate experiments. **P<0.01, ***P<0.001; two-tailed unpaired Student's *t*-test. Error bars indicate s.d. Cells treated with DMSO were used as a negative control. (C-E) Effect of SIPA1 on the transcription of the genes including *TGFB1* (TGFβ), *CTNNB1* (β-catenin), *SNAI1*, *SNAI2*, *ZEB1*, *CDH1*, *CDH2* and *VIM* in breast cancer cells. The mRNA level was determined through qRT-PCR in parent and SIPA1 knockdown MDA-MB-231 cells (C), parent and SIPA1 knockdown BT-549 cells (D), and parent and SIPA1-overexpressed MCF7 cells (E). Each column represents the mean of five separate experiments. Error bars indicate s.d. *P<0.05, **P<0.01, ***P<0.001; ns, no significance; two-tailed unpaired Student's *t*-test.

As described above, treating MCF7 cells with 5-Aza-CdR increased the expression of SIPA1, and a high level expression of SIPA1 resulted in EMT in MCF7 cells. In order to examine whether or not 5-Aza-CdR treatment led to the transition between epithelial and mesenchymal status in breast cancer cells, we treated MCF7 cells with various concentrations of 5-Aza-CdR and analyzed the expression level of E-cadherin and vimentin. As shown in Fig. 6A, the expression of E-cadherin was decreased with increasing the concentration of 5-Aza-CdR, whereas SIPA1 and vimentin expression was increased in a 5-Aza-CdR concentration-dependent manner. We next examined the effect of 5-Aza-CdR on the migration of MCF7 cells through a conventional transwell assay system. When MCF7 cells were treated with 5-Aza-CdR, the cell migration was facilitated in a concentration-dependent manner, as shown in Fig. 6B. To clarify the effect of 5-Aza-CdR on *VIM* and *CDH1*, we examined the protein expression in MDA-MB-231 cells after treatment with various concentrations of 5-Aza-CdR by western blotting. Interestingly, the expression of these proteins was not altered by 5-Aza-CdR treatment: vimentin expression was consistently high and E-cadherin was not detected (Fig. S5A). Furthermore, treating SIPA1-knockdown MDA-MB-231 cells with 5-Aza-CdR did not change the expression levels of SIPA1, vimentin and E-cadherin. In addition, there was no alteration in the migration activity, confirming that 5-Aza-CdR had no direct effect on the expression of *VIM* and *CDH1* in breast cancer cells with a low level of SIPA1 expression (Fig. S5A,B). Taken together, hypomethylation of *Sipa1* promoter-proximal elements by treating MCF7 cells with 5-Aza-CdR upregulated the expression of SIPA1 and vimentin, suppressed E-cadherin expression, and promoted the cancer cell migration, which might lead to EMT.

It has been demonstrated that signaling molecules, such as the transforming growth factor β (TGFβ) superfamily, the WNT family proteins (e.g. β-catenin) and the fibroblast growth factor family factors, could initialize and regulate the process of EMT by regulating the expression of key transcription factors, such as SNAIL1, SNAIL2 and ZEB1 (Dongre and Weinberg, 2019). We thus examined the relationship between the mRNA expression levels of *TGFB1* (TGFβ), *CTNNB1* (β-catenin), *SNAI1*, *SNAI2* and *ZEB1* and those of *Sipa1* in TCGA breast cancer patient samples. As shown in Fig. S6, the mRNA levels of *Sipa1* were positively correlated with those of *TGFB1*, *SNAI1* and *SNAI2*, but inversely correlated with *CTNNB1* transcription. Next, we examined the transcription levels of *Sipa1*, *TGFB1*, *CTNNB1*, *SNAI1*, *ZEB1*, *SNAI2*, *CDH1*, *CDH2* and *VIM* through qRT-PCR in breast cancer cells with different expression levels of SIPA1 (Fig. 6C-E). In SIPA1-knockdown MDA-MB-231 cells, *ZEB1* expression was decreased, and the expression of *CTNNB1* and *SNAI1* was increased (Fig. 6C), whereas no significant relationship between *Sipa1* and *CTNNB1* was observed in BT549 and MCF7 cells. Instead, *TGFB1* expression was decreased in SIPA1-knockdown BT549 cells (Fig. 6D), whereas the expression of both *ZEB1* and *TGFB1* was significantly increased in SIPA1-overexpressed MCF7 cells (Fig. 6E). These results demonstrate that a high level of SIPA1 expression could facilitate EMT by regulating the expression of differential EMT-related genes in breast cancer cells.

## DISCUSSION

Promoter hypomethylation in cancer cells has received less attention than hypermethylation, partly owing to the genome-wide loss of methylation in cancer and a low frequency of promoter hypomethylation in contrast to a high frequency of promoter hypermethylation. The present study, however, demonstrated that hypomethylation of the *Sipa1* promoter-proximal elements resulted in a high level of *Sipa1* transcription and SIPA1 protein expression in several cancer cells. Because normal epithelial cells in the breast duct hardly activate *Sipa1*, the transcription of *Sipa1* via promoter hypomethylation seems to be an aberrant event associated with cancer progression. In fact, the present findings demonstrated that SIPA1 contributed to the maintenance of EMT in MDA-MB-231 cells. In addition, it is likely that *Sipa1* hypomethylation also facilitates the migration of other types of cancer cells, such as colorectal and prostate cancer cells (Dongre and Weinberg, 2019).

The mechanism underlying *Sipa1* promoter hypomethylation in metastatic cancer cells has not been elucidated yet. It is thus unclear whether *Sipa1* promoter hypomethylation is associated with genome-wide alterations in methylation status or is an independent gene-specific event. Present data show that the methylation level of the *Sipa1* promoter-proximal elements in individual cell lines is dependent on cell lines and highly heterogeneous. The variation, therefore, might reflect stochastic noise or dynamic methylation processes during cancer progression and prognosis. The methylation status of the *Sipa1* promoter, however, exhibits locus specificity: a comparatively reduced level of methylation in the promoter region within the 10th to 16th CpG motifs was observed in several cell lines. It is thus possible that the methylation of *Sipa1* CpGs is regulated in a sequence-specific manner.

Despite the distinct evidence for hypomethylation-linked activation of *Sipa1*, the precise mechanism by which *Sipa1* promoter is regulated has not yet been deciphered. The present study shows that DNA methylation status has a substantial influence on tumor progression by regulating oncogenes such as *Sipa1*. Demethylation agents, such as 5-Aza-CdR and 5-Aza-cytidine have been used as chemotherapeutic agents in the treatment of malignancies such as myeloid leukemia (Garcia-Manero et al., 2006; Kornblith et al., 2002). The therapeutic efficacy of these agents is attributed to their inhibitory activity against DNA methyltransferases and their cytotoxic effects. Based on the present data, the inhibitors could facilitate the transcription of certain oncogenes such as *Sipa1*, which might have influence on the fate of cancer cells. 5-Aza-CdR treatment is, however, not specific to the *Sipa1* promoter in MCF7 cells, and the other oncogenes and tumor suppressor genes might also be upregulated. Whereas treatment with 5-Aza-CdR facilitated the migration of MCF7 cells in a concentration-dependent manner, it was not clear why E-cadherin expression was decreased in MCF7 cells after 5-Aza-CdR treatment. To address this issue, we analyzed the methylation status of *CDH1* gene coding E-cadherin based on previous reports, and found that the *CDH1* promoter region was mostly in a hypomethylation status in tumor specimens from breast cancer patients and MCF7 cells (Terada et al., 2009; Huang et al., 2019), suggesting that E-cadherin expression is not simply regulated by the methylation status of *CDH1*. It is thus crucial to consider the possibility that other pivotal genes might also be regulated after treatment of tumor cells with 5-Aza-CdR, besides the expression of SIPA1 and E-cadherin. We also have to pay attention to the long-term risks and side effects when using demethylation agents for cancer therapy, and personalized treatment should be optimized based on the methylation status of genes within the tumors of individual patients.

## MATERIALS AND METHODS

### Cell lines, culture conditions

The human breast cancer cell lines MDA-MB-231 and MCF7, and the human colon cancer cell lines Caco2 and SW480 were purchased from and authenticated by the China Center for Type Culture Collection (CCTCC, Wuhan, Hubei, China). The human breast cancer cell line SK-BR3 and the human prostate cancer cell lines PC3 and LNCaP were purchased from Xiangfa Bio (Minhang, Shanghai, China) with short tandem repeat (STR) authentication. The human endometroid adenocarcinoma cell line HEC1A was obtained from the Department of Pathology of the Hubei Cancer Hospital (Wuhan, Hubei, China) and authenticated by STR analysis at CCTCC. The human breast cancer cell line BT549 and the human colon cancer cell line HCT116 were purchased from Procell Life Science and Technology (Wuhan, Hubei, China) with STR authentication. The *Sipa1*-knockdown MDA-MB-231 cell line (MDA-MB-231/sh-*Sipa1*) and the *Sipa1*-knockdown BT549 cell line (BT549/sh-*Sipa1*) were established following the previous study (Zhang et al., 2015). All the cell lines were maintained in DMEM or RPMI-1640 medium (Thermo Fisher Scientific) supplemented with 10% fetal bovine serum, 50 IU ml^−1^ penicillin and 50 μg ml^−1^ streptomycin, with 5% CO_2_ at 37°C.

For 5-Aza-2′-CdR (Sigma) treatment, a 10 mM stock solution was prepared by dissolving the compound in DMSO, which was then diluted in cell culture medium to the indicated concentrations. Cells were cultured in the medium supplemented with DMSO for 3 consecutive days for a negative control.

### Western blotting

Cells were detached with 0.25% trypsin/1 mM Na_2_EDTA and collected in tubes. After centrifugation, the cell pellets were dissolved in lysis buffer [10 mM HEPES (pH 7.9), 500 mM NaCl, 0.1 mM EDTA, 0.1 mM EGTA, 0.1% NP-40, 1 mM PMSF and 1% protease inhibitor cocktail] on ice for 10 min, and the protein concentrations were determined using the standard Bradford reagent. Samples with equal amounts of protein (20 μg) were resolved by SDS-PAGE on 8-12% gels and subsequently transferred to nitrocellulose membranes (Millipore), which were then submerged in blocking buffer (5% nonfat dry milk in TBS and 0.1% Tween 20) for 1 h at room temperature. Membranes were then incubated with a 1:1000 dilution of a primary antibody against SIPA1 (189929; Abcam), E-cadherin (14472S; Cell Signaling Technology), vimentin (5741S; Cell Signaling Technology), GAPDH (GB11002; Servicebio) or β-actin (AC004; ABclonal) followed by a 1:20,000 dilution of HRP-linked anti-rabbit or anti-mouse secondary antibody (7074 and 7076; Cell Signaling Technology). The signals were visualized using ECL Western Blotting Substrate (Thermo Fisher Scientific).

### Luciferase assay

DNA fragments were cloned into the pGL-4 basic luciferase expression vector (Promega) as described previously (Zhang et al., 2015). The plasmids and the pRL-TK plasmid were co-transfected into HEK293 or MDA-MB-231 cells. Reporter assays were performed in cells transfected with the indicated promoter constructs and analyzed using the Dual-Luciferase Reporter Assay Kit (Promega). The luciferase activity was measured by a GloMax 20/20 luminometer (Promega) and then normalized to the value for the co-transfected Renilla plasmid. All the data were processed using a GraphPad 5.0 software.

### DNA methylation

For hypermethylation, the plasmids with or without promoter candidates were treated with CpG methyltransferase *M.Sss*I (New England Biolabs) and 3mM S-adenosylmethionine (SAM, Sigma) at 37°C for 4 h. After heat inactivation of the enzyme at 65°C for 20 min, plasmids were purified for the methylation-sensitive digestion assay and the luciferase activity assay.

### Methylation-sensitive digestion assay

To examine the methylation status of the pGL-4 plasmids with or without promoter candidate fragments after the enzymatic methylation by *M.Sss*I, plasmids were digested with methylation-sensitive restriction enzyme HpaI or HhaI (New England Biolabs) and digestion products were separated by 2% agarose gel electrophoresis and stained for imaging.

### DNA demethylation with 5-Aza-2′-CdR

For 5-Aza-2′-CdR (Sigma) treatment, a 10 mM stock solution was prepared by dissolving the compound in DMSO, which was then diluted in cell culture medium to the indicated concentrations. Cells were cultured in the medium supplemented with DMSO for 3 consecutive days for a negative control.

### Transient plasmid transfection

Transient overexpression of the genes of interest was conducted using Lipofectamine 2000 (Invitrogen) according to the manufacturer's instructions. Transformation by electroporation was performed using a Gene Pulser Xcell machine (Bio-Rad) following the manufacturer's instructions.

### RNA extraction and quantitative real-time PCR (qRT-PCR)

Total RNA was extracted from cultured cancer cells using TRIzol reagent (Invitrogen) according to the manufacturer's protocol. Equal amounts of RNA were used for the synthesis of first-strand cDNA. One microliter of the reverse-transcribed product was used as a template to amplify the genes of interest with specific primers, which were synthesized according to the sequences from the Website of Primer Bank (pga.mgh.harvard.edu/primerbank/) (Table S4). Real-time PCR amplification was conducted using an Applied Biosystems StepOne real-time PCR system, and relative fold changes were determined using the 2^−ΔΔCt^ method, in which GAPDH was used for normalization.

### Methylation-specific PCR (MSP) and bisulfite sequencing PCR (BSP)

Genomic DNA was extracted from cultured cell lines, and the concentration was determined using a NanoDrop2000 spectrophotometer. Five hundred nanograms of DNA sample were diluted with double-distilled H_2_O to 20 μl. The genomic DNA samples were then treated with sodium bisulfite using the EZ DNA Methylation-Gold Kit (Zymo Research) according to the manufacturer's instructions and prepared for MSP or BSP. For MSP, a pair of primers to amplify only methylated CpG targets were designed based on the CpG island sequence of the *Sipa1* promoter-proximal elements, and the sequences were as follows: 5′-CGTTGTTGCGTTTTTCGTC-3′ (forward) and 5′-AATACTAACGACGAACGTAACG-3′ (reverse). Another pair of primers, to amplify unmethylated CpG targets, were designed as follows: 5′-TTTGTTGTTGTGTTTTTTGTT-3′ (forward) and 5′-TGTTATGTTTGTTGTTAGTATTTTTTT-3′ (reverse). MSP was performed via a standard PCR machine, and the PCR products were resolved by agarose gel electrophoresis and stained with ethidium bromide. For BSP, the following specific primers were synthesized: 5′-GAAATGGTTTTAGAATGAGTATTGTT-3′ (forward) and 5′-CTAAACCACCTCTCCCCTAT-3′ (reverse). The PCR products were recycled and inserted into the T vector. Then, the ligation products were transfected into DH5α competent cells. After culturing at 37°C for 16 h, single colonies were picked and cultured, and then the plasmid was purified and sequenced by the Tsingke company. Sequences from ten clones of each sample were then analyzed using a BiQ Analyser software (Bock et al., 2005). The methylation level of each CpG dinucleotide was calculated as the ratio of the number of positive clones (methylated CpG dinucleotide) to the total number of clones examined (eight).

### Transwell assay

Cell migration was analyzed *in vitro* using Transwell permeable supports (Corning). In brief, cells were seeded into each insert of the 24-well plate and incubated at 37°C for 24 h, except HCT116 and Caco2 cells, which were incubated for 40 h. After removal of the cells on the top of the membrane with cotton swabs, the cells on the bottom of the membrane were fixed with methanol for 10 min, followed by staining with Crystal Violet for 5 min. After washing with distilled water, the migrated cells on the membrane were counted in five random fields of view in each membrane, and images were captured by a microscope containing a CCD camera (Motic).

### Immunofluorescence

Immunostaining was performed as previously described (Hsu et al., 2012). In brief, cells were seeded onto cover glasses placed in 24-well plates, and incubated with primary antibodies overnight at 4°C, followed by staining with TRITC- or rhodamine-coupled secondary antibodies (Protein Tech Group) for 1 h at room temperature. Cells were visualized by laser scanning microscopy (Olympus IX71) and the images were analyzed using Andor IQ 2 software (www.andor.com).

### Bioinformatics prediction and correlation analysis

For *Sipa1* promoter prediction, possible regions were selected according to the prediction result in the SwitchGear LightSwitch Promoter Reporter GoClone collection (switchgeargenomics.com/products/promoter-reporter-collection). Four additional online programs, i.e. Promoter 2.0 Prediction (www.cbs.dtu.dk/services/Promoter/), McPromoter (tools.genome.duke.edu/generagulation/McPromoter), Neural Network Promoter Prediction (www.fruitfly.org/seq_tools/promoter.html) and PROSCAN Version 1.7 (www-bimas.cit.nih.gov/molbio/proscan/), were used to further evaluate the promoter potential. CpG island prediction was performed online with CpGPlot (emboss.bioinformatics.nl/cgi-bin/emboss/cpgplot) and MethPrimer (www.urogene.org/cgi-bin/methprimer/methprimer.cgi). For correlation analyses, the methylation level of *Sipa1* gene and the transcription data of *Sipa1*, *VIM* and *CDH1* in breast cancer tumors were downloaded from TCGA breast cancer tissues datasets (www.cancer.gov/tcga) (Ciriello et al., 2015). The correlation analysis was performed using a scatterplot. A linear regression analysis was performed using a GraphPad 6.0 software to evaluate the correlation between two factors.

## Supplementary Material

Supplementary information
