# Supplementary material for: 5-Aza-2′-deoxycytidine advances the epithelial–mesenchymal transition of breast cancer cells by demethylating Sipa1 promoter-proximal elements
Source: J Cell Sci. 2020 May 11;133(9):jcs236125. doi: 10.1242/jcs.236125 (PMC7240297; doi:10.1242/jcs.236125)
Supplement: Supplementary information [file joces-133-236125-s1.pdf]

Supplementary Information

Table S1. Possible promoter sequences predicted by the software Promoter 2.0.

Segment	Position	Score*	Prediction
SP-1093	700-800	0.692	Likely
SP-767	–	–	–
SP-1167	600-700	0.533	Likely

* A higher score represents a larger possibility of the sequence as a promoter and vice versa.

Table S2. Possible promoter sequences predicted by the software Berkeley Drosophila Genome Project.

Segment	Position	Score	Predicted sequence
SP-1093	626-676	0.93	TTTTAGAATAAATGAGAGCGCGCTAG TGGAATGGTTGCCCAGGGCCCAGT
SP-1093	683-733	0.98	AAGTGCTCCATAAAATAGTGGCAGAC TCTTGAGCACCTGCTGGCCGGGAG
SP-767	–	–	–
SP-1167	348-398	0.69	GGCCTCCCAAAGTGCTGGGATTACAG GCCACAGCCACCGCACCCGGCCAG

Table S3. Possible promoter sequences predicted by Proscan (ver. 1.7).

Segment	Direction	Position	Score	Promoter cutoff
SP-1093	Forward	701-951	71.55	53.0
SP-767	Forward	197-447	94.84	53.0
SP-1167	Forward	–	–	–

Journal of Cell Science • Supplementary information

Table S4. List of Primers for qRT-PCR

Primers	Sequences
Sipa1-Forward	CCTTCATGCAGTTTCTCACCTTGC
Sipa1-Reverse	GTGGTCCTGGTATGTGGTGTAGAG
GAPDH-Forward	TGAACGGGAAGCTCACTGG
GAPDH-Reverse	TCCACCACCCTGTTGCTGTA
ZEB1-Forward	TTACACCTTTGCATACAGAACCC
ZEB1-Reverse	TTTACGATTACACCCAGACTGC
SNAI1-Forward	ACTGCAACAAGGAATACCTCAG
SNAI1-Reverse	GCACTGGTACTTCTTGACATCTG
CDH1-Forward	ATTTTTCCCTCGACACCCGAT
CDH1-Reverse	TCCCAGGCGTAGACCAAGA
VIM-Forward	AGTCCACTGAGTACCGGAGAC
VIM-Reverse	CATTTCACGCATCTGGCGTTC
TGFB1-Forward	CTAATGGTGGAAACCCACAACG
TGFB1-Reverse	TATCGCCAGGAATTGTTGCTG
SNAI2-Forward	CGAACTGGACACACATACAGTG
SNAI2-Reverse	CTGAGGATCTCTGGTTGTGGT
CTNNB1-Forward	CATCTACACAGTTTGATGCTGCT
CTNNB1-Reverse	GCAGTTTTGTCAGTTCAGGGA
CDH2-Forward	AGCCAACCTTAACTGAGGAGT
CDH2-Reverse	GGCAAGTTGATTGGAGGGATG

Journal of Cell Science • Supplementary information

Figure S1. The constructs derived from SP-1093 and detection of their transcriptional activity. (A) Schematic diagram of extension and deletion constructs derived from SP-1093. These constructs are prepared to identify the regions that facilitate the *Sipa1* transcription. **(B) Transcriptional activity of SP-456, SP-656, SP-856, SP-1093 and SP-1203, as assessed through a luciferase assay.** The respective plasmids and pGL-4 as a control were co-transfected into HEK293 cells with pRL-TK. Each column represents the mean of triplicate experiments (ns, no significance; two-tailed unpaired Student's *t*-test. Error bars = s.d.).

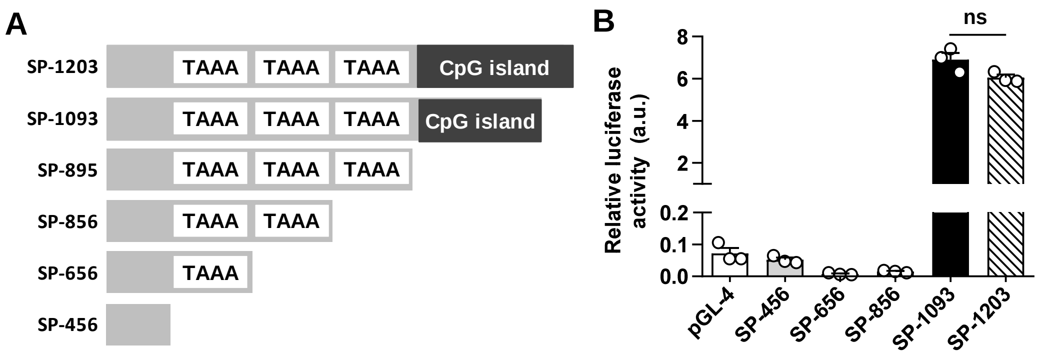

Journal of Cell Science • Supplementary information

Figure S2. Methylation status of the *Sipa1* CpG islands in 10 cancer cell lines revealed by the bisulfite sequencing PCR (BSP). The short vertical lines indicate 30 individual CpG dinucleotides. Methylated and unmethylated cytosines are indicated by black and white squares, respectively.

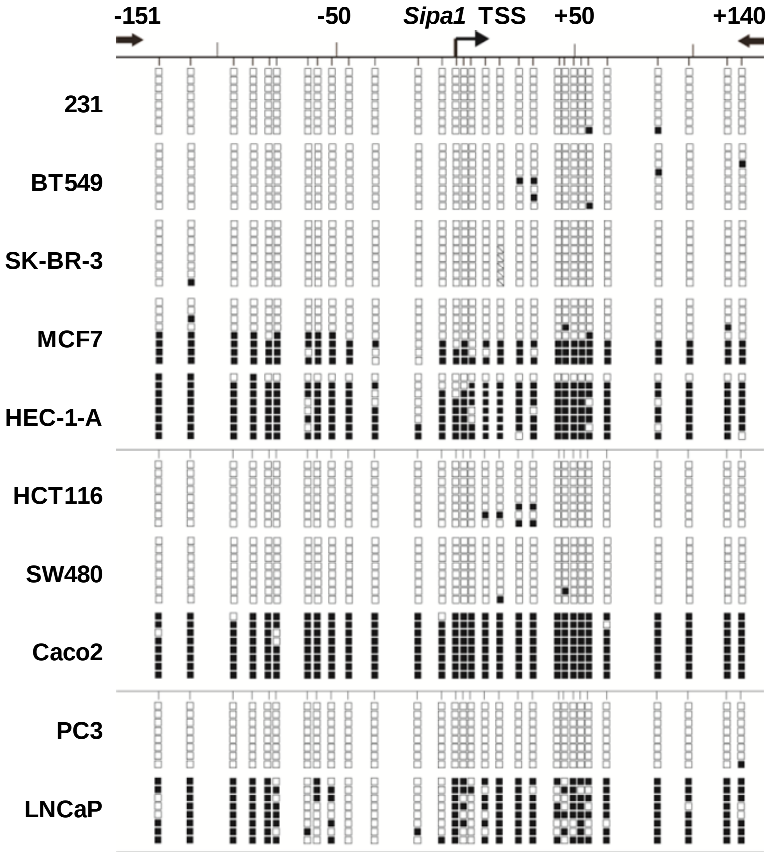

Journal of Cell Science • Supplementary information

Figure S3. Methylation status of the *Sipa1* promoter region CpG islands and *Sipa1* transcription levels in human tissues and cancer cell lines. Heatmap depicting the proportion of methylated CpG dinucleotides within the *Sipa1* CpG island in human tissues (A) and two cancer cell lines (C). Data were extracted from the GEO whole-genome bisulfite sequencing data in human tissues and cancer cell lines. Each box indicates one of 30 individual CpG dinucleotides. The proportion is represented by the darkness of the red color, and the methylation ranged from 0% to 100%. **Transcription levels of *Sipa1* in human tissues (B) and two breast cancer cell lines (D).** Data were obtained from the website of Human Protein Atlas (https://www.proteinatlas.org/).

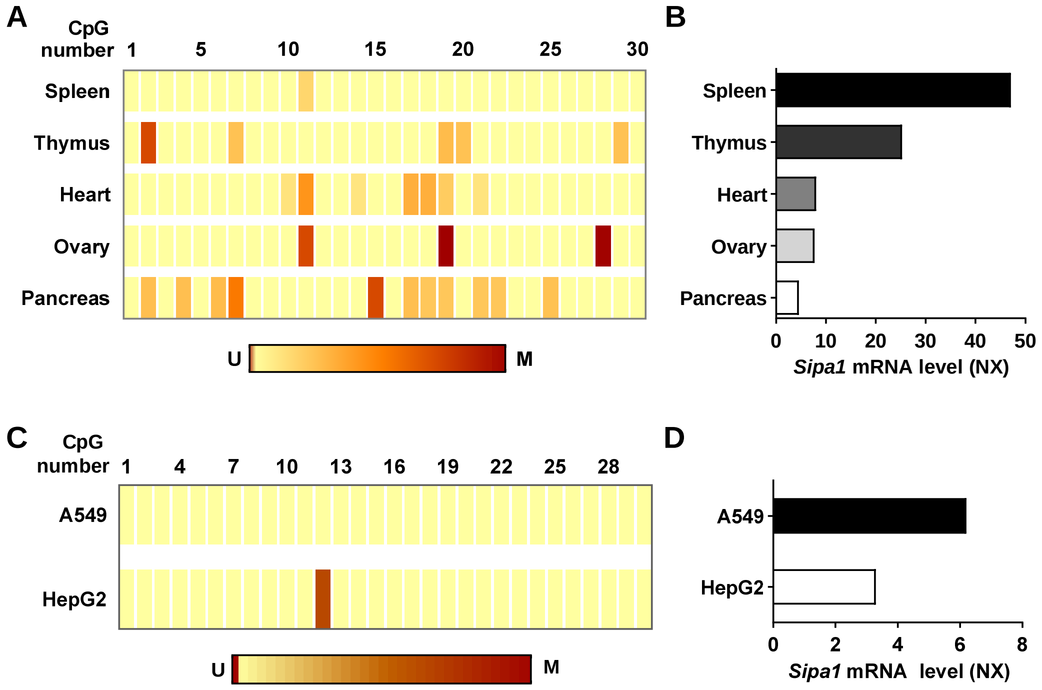

Journal of Cell Science • Supplementary information

Figure S4. The expression of SIPA1, E-cadherin and vimentin in BT549 cells over-expressing SIPA1. The cells were transfected with pcDNA3-*Sipa1* plasmid, and then analyzed by Western blotting.

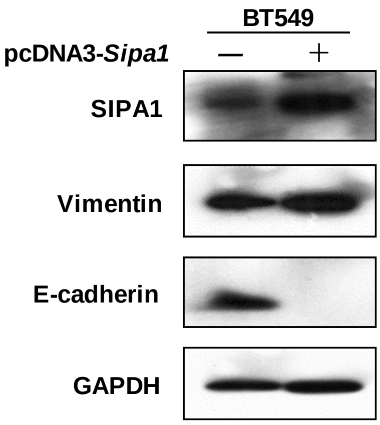

Journal of Cell Science • Supplementary information

Figure S5. Effect of 5-Aza-CdR on the expression of SIPA1, E-cadherin, and vimentin and on the migration of parent and SIPA1-knockdown MDA-MB-231 cells. (A) Effect of 5-Aza-CdR on the expression of SIPA1, E-cadherin, and vimentin in MDA-MB-231 and MDA-MB-231/sh-*Sipa1* cells revealed by Western blotting. MCF7 cell sample was loaded as a positive control for E-cadherin expression. **(B) Effect of 5-Aza-CdR on the migration of MDA-MB-231/sh-*Sipa1* cells.** The cells were treated with different concentrations of 5-Aza-CdR in transwells and the migrated cells were counted. Each column represents the mean of triplicate experiments (ns, no significance; two-tailed unpaired Student's *t*-test. Error bars = s.d.).

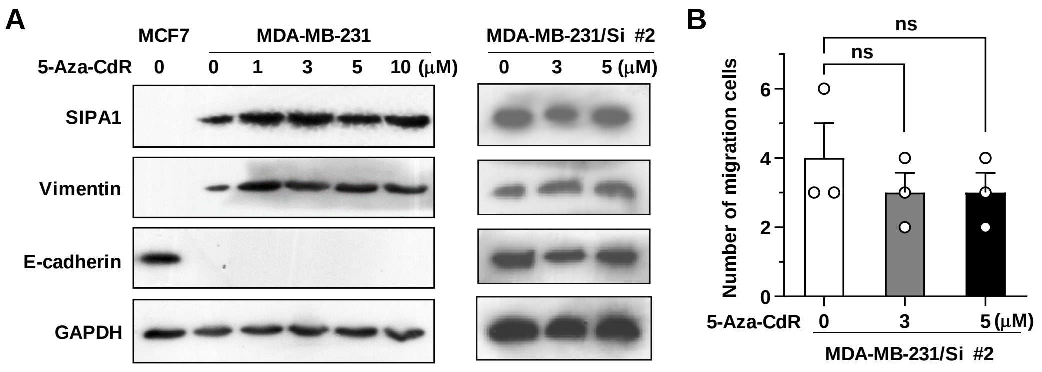

Journal of Cell Science • Supplementary information

Figure S6. Correlation between the mRNA expression of *Sipa1* and that of *TGFB1*, *CTNNB1*, *SNAI1* or *SNAI2* in breast cancer cells. A linear regression analysis was conducted using the gene expression data in human breast tumor samples from TCGA breast cancer tissues data set.

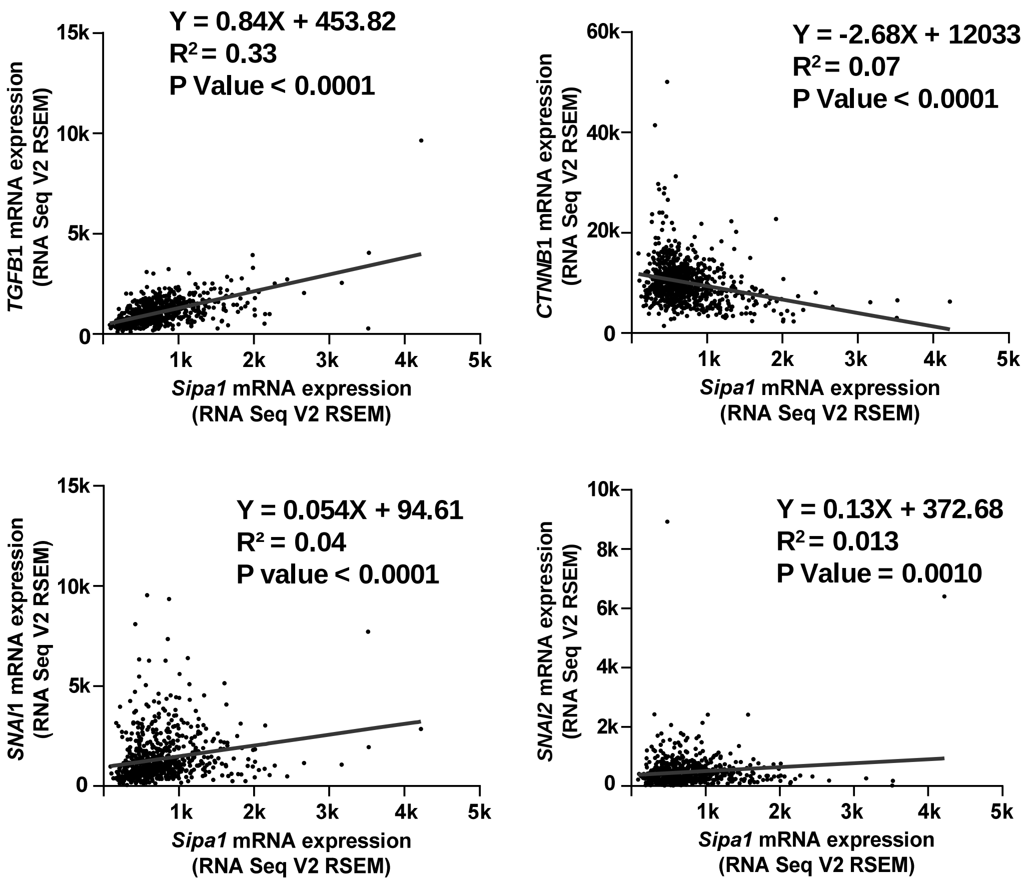

Journal of Cell Science • Supplementary information
